# Expression of matrix metalloproteinases (MMPs) in primary human breast cancer and breast cancer cell lines: New findings and review of the literature

**DOI:** 10.1186/1471-2407-9-188

**Published:** 2009-06-16

**Authors:** Andrea Köhrmann, Ulrike Kammerer, Michaela Kapp, Johannes Dietl, Jelena Anacker

**Affiliations:** 1 Department of Obstetrics and Gynecology, University of Würzburg, Josef-Schneider Str. 4, 97080 Würzburg, Germany

## Abstract

**Background:**

Matrix metalloproteinases (MMPs) are a family of structural and functional related endopeptidases. They play a crucial role in tumor invasion and building of metastatic formations because of their ability to degrade extracellular matrix proteins. Under physiological conditions their activity is precisely regulated in order to prevent tissue disruption. This physiological balance seems to be disrupted in cancer making tumor cells capable of invading the tissue. In breast cancer different expression levels of several MMPs have been found.

**Methods:**

To fill the gap in our knowledge about MMP expression in breast cancer, we analyzed the expression of all known human MMPs in a panel of twenty-five tissue samples (five normal breast tissues, ten grade 2 (G2) and ten grade 3 (G3) breast cancer tissues). As we found different expression levels for several MMPs in normal breast and breast cancer tissue as well as depending on tumor grade, we additionally analyzed the expression of MMPs in four breast cancer cell lines (MCF-7, MDA-MB-468, BT 20, ZR 75/1) commonly used in research. The results could thus be used as model for further studies on human breast cancer. Expression analysis was performed on mRNA and protein level using semiquantitative RT-PCR, Western blot, immunohistochemistry and immunocytochemistry.

**Results:**

In summary, we identified several MMPs (MMP-1, -2, -8, -9, -10, -11, -12, -13, -15, -19, -23, -24, -27 and -28) with a stronger expression in breast cancer tissue compared to normal breast tissue. Of those, expression of MMP-8, -10, -12 and -27 is related to tumor grade since it is higher in analyzed G3 compared to G2 tissue samples. In contrast, MMP-7 and MMP-27 mRNA showed a weaker expression in tumor samples compared to healthy tissue. In addition, we demonstrated that the four breast cancer cell lines examined, are constitutively expressing a wide variety of MMPs. Of those, MDA-MB-468 showed the strongest mRNA and protein expression for most of the MMPs analyzed.

**Conclusion:**

MMP-1, -2, -8, -9, -10, -11, -12, -13, -15, -19, -23, -24, -27 and -28 might thus be associated with breast cancer development and tumor progression. Therefore, these MMPs are proper candidates for further functional analysis of their role in breast cancer.

## Background

Breast cancer is the most common cancer affecting women in the world today. It is the leading cause of cancer related death for women aged between 35 and 55 years worldwide. One in nine women will suffer from breast cancer during her life and in excess 130 thousand women die from breast cancer each year [1]. According to histological features invasive breast cancers are classified into three groups: well differentiated (grade 1, G1), moderately differentiated (grade 2, G2) and poorly differentiated (grade 3, G3) tumors. Distant metastases are the principal cause of death. An essential process in forming distant metastases is the degradation of the extracellular matrix allowing tumor cells to invade local tissue, intravasate and extravasate blood vessels and build new metastatic formations. This process is primarily influenced by the activity of proteinases secreted by the tumor. Currently, at least four classes of proteinases are known: serine proteinases, aspartatic proteinases, cystein proteinases and matrix metalloproteinases [2-4]. Collectively, these proteinases are capable of breaking down all components of the extracellular matrix. Under physiological conditions (e.g. tissue remodeling, angiogenesis, ovulation, wound healing) there is a precise regulation between proteolytic degradation and regulatory inhibition of proteolysis [2-5]. This physiological balance seems to be disrupted in cancer. Matrix metalloproteinases (MMPs) are up regulated in almost every type of cancer and their expression is often associated with a poor prognosis for patients [6,7]. Previous studies have shown the expression and activity of MMPs to be linked to an advanced stage of breast cancer, increased invasion of tumour cells and building of metastatic formations [reviewed in [8]].

MMPs are a family of structural and functional related endopeptidases. They are, with exception of MMP-11, secreted as inactive zymogens and activated outside the cell by other activated MMPs or serine proteases (e.g trypsin, plasmin, kallikrein) [2-4]. For their activation, a proteolytic removal of the propeptide-domain is required. This enables access to the catalytic site of the MMPs. The cleavage of the extracellular matrix (ECM) by activated MMPs facilitates the invasion of tumor cells as well as the release of ECM bound growth factors (e.g. of insulin like growth factors and fibroblast growth factors). Further, some of the resulting ECM-protein fragments can feature new biological functions (e.g. cleavage of laminin-5 or collagen type IV results in uncovering of their cryptic site which can promote migration of different cell types) [2-4].

Currently, 23 members of the MMP family are known in humans. According to their substrate specificity, they are divided into six subclasses: collagenases, gelatinases, stromelysins, matrilysins, membrane-type MMPs and others [2]. So far, most investigators have focused on the expression profile of the two gelatinases, MMP-2 and MMP-9, which are able to degrade type IV collagen. [9-11]. Type IV collagen is abundant in basement membranes separating the epithelial cells from the underlying stroma. Increased expression and activity of MMP-2 and -9 in tumors leads to the degradation of basement membranes, an essential step in tumor invasion. In this respect, a correlation between a high expression of MMP-2 and reduced survival in breast cancer patients [12] as well as an association of the tumor grade with increased levels of MMP-9 in breast cancer tissue [13] was described. Efficient reduction of MMP-2 and -9 levels was observed during *in vitro *treatment of MCF-7 breast cancer cells with the aromatase-inhibitor letrozole suggesting that this inhibitor suppresses both breast cancer growth and invasion [14].

For MMP-1, -7, -9, -13 and -14 an association between their high expression and a shortened relapse free survival in breast cancer patients was found [15]. A high expression of MMP-9 and -11 in breast cancer tissue was also detected by Northern blot analysis [16]. Vizoso et al. showed a correlation between high expression levels of these two MMPs and a higher rate of distant metastases using immunohistochemistry [15]. In addition, the expression of MMP-2, -8, -9, -10, -11 and -13 mRNA in breast cancer tissue was identified by RT-PCR [17]. Gonzalez et al. found MMP-1, -7 and -13 to be expressed at higher levels in androgen receptor positive breast cancer cells using immunohistochemistry [18]. This suggests, that androgen receptors might be able to up regulate MMPs and contribute to a higher invasive potential of breast cancer cells. Using Northern Blot analysis, a higher expression of MMP-2, -7, -9 and -11 mRNA was shown in breast cancer tissue in comparison to normal breast tissue [19]. In addition, a higher content of MMP-1 and -9 protein was detected in breast cancer tissue when compared to normal breast tissue by ELISA technique [20]. Using substrate zymography, MMP-1, -2, -3 and -9 demonstrated a higher activity in tumor tissues compared to healthy samples [5]. Expression of MMP-3 was shown to be higher in stromal tissue surrounding the epithelial tumor cells than in tumor cells themselves [21]. A correlation between positive nodal status and the expression of MMP-14 and -15 mRNA was found by Ueno et al. [22]. Haupt et al. found MMP-14 to be expressed predominantly in preinvasive lesions of breast cancer using RT-PCR [21]. To our knowledge, currently there are no data available for the remaining MMPs regarding their expression in breast cancer tissue in literature.

Thus, the aim of our study was to investigate, if a pattern of MMPs could be identified, whose expression is related to tumor grade in breast cancer. With this objective, we analyzed the expression of all human MMPs known so far in a panel of normal breast and breast cancer samples by semiquantitative RT-PCR, Western Blot and immunohistochemistry. Further, in order to create an independent and reproducible model system for the in vitro analysis of the regulation of MMP expression in breast cancer cells, we analyzed the MMP-pattern in four breast cancer cell lines frequently used in basic breast cancer research (MCF-7, BT-20, MDA-MB-468, ZR 75/1). For an overview, data concerning MMP expression in breast cancer cell lines published so far are summarized in Additional file 1: MMP expression in different breast cancer cell lines [23-27].

## Methods

### Tissue samples

The study was performed with approval of the Ethics Committee of the University of Wuerzburg, Germany. To establish an expression profile of MMPs typical for breast cancer, we compared normal breast tissue to breast cancer tissue with higher grading (G2 and G3). We excluded well-differentiated breast cancer (G1) tissue, because material of G1-tumors was rare. MMP expression was analyzed in five normal breast and twenty breast cancer tissue samples. The normal breast tissue samples were obtained from patients who underwent reductive mammoplasty for cosmetic reasons (tissue was analyzed histologically to exclude that there were any forms of malignancy or other pathological findings; data not shown). Written informed consent was obtained from each patient according to our institutional regulations. Twenty samples of breast cancer tissue (ten G2 and ten G3 tumors) were obtained during surgical removal of the tumor at the department of Gynaecology and Obstetrics at the University Hospital of Wuerzburg, Germany. Some parts of the samples were immediately frozen in liquid nitrogen and stored at -80°C, other parts were fixed in formalin and embedded in paraffin. Patients' data are listed in Table 1. Tissue was selected in order to contain approximately equal amounts of stromal tissue (as analyzed by HE-staining). Therefore stroma was present to nearly the same extent in each tissue sample that was analyzed by RT-PCR and Western blot analysis. Tissue samples were examined for content of benign and/or malign glandular tissue by HE-staining also. Only samples with high density of benign lobuli/acini or tumour cell mass without signs of necrosis were used for further analysis.

**Table 1:** Patients data

	G2-tumors (n = 10)	G3-tumors (n = 10)
age at diagnosis (y)		
mean	64,7	71,8
minimum	42	52
maximum	91	90

T (tumor size)		
1	4	3
2	5	2
3	-	-
4	1	5

N (nodal status)		
0	6	1
1	2	5
2	1	2
X	1	2

M		
0	10	6
1	-	4

L		
0	7	2
1	3	8

multifocal	4	3
inflammatory	1	4
invasive ductal	8	9
invasive ductal +DCIS	2	-
invasive lobular	-	1

receptor status		
ER +/PR+	3	4
ER +/PR -	1	1
ER -/any PR	5	3
n.d.	1	2

Her2neu 2+/3+	2	3
Her2neu negative	5	2
n.d.	3	5

G1 = well differentiated tumor; G2 = moderatly differentiated tumor; G3 = poorly differentiated tumor (the three-tier grading system (G1-3) is usually used at our institution to determine the histologic grade of tumor tissue). T = tumor grade; N = nodal status; M = distant metastasis; L = invasion of lymphatic vessels; DCIS = ductal carcinoma in situ; ER = estrogen receptor; PR = progesterone receptor; n.d. = not done.

### Cell culture

Cell lines (MCF-7, MDA-MB-468, BT 20, ZR 75/1) were obtained from Cell Lines Service (Eppelheim, Germany) [28]. Characteristics of the cell lines are listed in Table 2[29-36]. Briefly, cells were cultured in a mixture of DMEM/Ham's F-12 (PAA, Coelbe, Germany) supplemented with 10% FCS and 10 ng/ml gentamycine at 37°C in the presence of 5% CO_2_. Cells were cultured in 75 ml culture-flasks (Biochrom, Berlin, Germany) as monolayer culture and harvested at 80–90% confluency using a cell-scraper (Biochrom) or accutase (PAA) treatment. Separated cells were resuspended in phosphate-buffered saline (PBS) and washed twice. Cells were then counted and checked for viability using trypan blue and either subjected to immunocytochemistry or immediately frozen as dry pellets at -20°C for further analysis. All cell preparations used had a viability of >95%.

**Table 2:** Cell lines

Cell line	MCF-7	MDA-MB 468	BT 20	ZR 75/1
Cell type	Adeno-carcinoma	Adeno-carcinoma	Invasive ductal carcinoma	Adeno-carcinoma

Origin	Metastasis (pleural effusion)	Metastasis (pleural effusion)	Primary tumor	Metastasis (ascites fluid)

Estrogen-/progesterone-receptor	+/+	-/-	-/-	+/+

Invasive potential	Low	Low	Low	-

Metastatic potential	-/+	Low	-	-

Table based on data published previously [29-36]

### RNA extraction and cDNA synthesis

Frozen blocks (1 cm^2^) of normal and tumor tissue were cut into sections of 6 μm; sections corresponding to 30 mg were collected in a sterile microtube and subjected to RNA isolation. In case of cultured cell lines, 10^6 ^cells were used for RNA extraction. Total RNA was extracted using RNeasy mini kit (Qiagen, Hilden, Germany) according to the manufacturers' instruction. RNA was eluted in 60 μl RNase free water and stored at -20°C. Total RNA was reverse transcribed at 42°C for 1 h in a 20 μl reaction mixture using the RevertAid H Minus First Strand cDNA synthesis kit (Fermentas, St. Leon-Roth, Germany) and terminated by heating the samples at 70°C for 10 min. Synthesized cDNA was stored at -20°C for further expression analysis.

### Semiquantitative RT-PCR

Expression analysis of MMPs was performed using self-created gene specific primers. Primer sequences and PCR conditions are summarized in Additional File 2. In general, conventional PCR reaction was performed in 25 μl volumes containing template DNA, 2.5 U Taq polymerase, 10× buffer with 1.5 mM MgCl_2 _(all Eppendorf, Hamburg, Germany), 200 μM dNTPs (Fermentas), 0.4 μM of both, forward and reverse primers and formamide, which was used optionally at a final concentration of 4%. PCR conditions were optimized for each primer-pair. Amplification reactions were performed using a Px2 thermal cycler (Techne, Staffordshire, U.K.) and consisted of following steps: 94°C for 5 min, 28–32 cycles at 94°C for 30 sec; optimized annealing temperatures for 30 sec and 72°C for 10 min. The amount of cDNA was normalized to the intensity of the PCR products of the housekeeping gene (PBGD) [37]. All PCR products were separated on 1% agarose gels and visualized using GelRed (Biotium, Inc., Hayward, CA). Intensity of GelRed luminescence was measured using ImageJ software (NIH, Bethesda, USA). All RT-PCRs were performed in triplicates.

### Western blotting

For protein extraction, 20 mg of cryo-cut tissue samples or respectively 10^6 ^cells were lysed in pre-cooled Ripa-buffer (Pierce, Rockford, Ilinois) containing phosphatase inhibitors (Phosphatase Inhibitor Cocktails Set II, Calbiochem, Germany), proteinase inhibitors (complete, Roche, Germany) and 2,5 mM DTT (Dithiothreitol, Sigma, Taufkirchen, Germany) as reducing agent. The mixture was incubated on ice for 30 min, combined with vortexing every 10 min. Cell lysates were clarified of cell debris by centrifugation at 14.000 g for 5 min through a QIAshredder spin column assembly (Qiagen, Hilden, Germany). Afterwards, the samples were mixed in 5× loading buffer (Fermentas), denatured at 95°C for 5 min, chilled on ice and stored at -20°C for further analysis.

Protein concentration was determined by the Bradford-method [38] using comassie brilliant blue (Roti-Quant; Roth, Karsruhe, Germay). Samples were subjected to electrophoresis on a 10% polyacrylamide gel (SDS-PAGE) and blotted onto a nitrocellulose membrane (Schleicher & Schuell, Dassel, Germany) for 45 min at 10 V using a semi-dry-transfer unit (PeqLab, Erlangen, Germany). Membranes were stained with ponceau-red to verify that the proteins were blotted. To avoid non-specific binding, membranes were blocked with 5% nonfat milk protein in PBS/Tween at RT for 1 hour. Subsequently, the membranes were incubated with the primary antibody diluted in 2% nonfat milk and PBS/Tween at 4°C for 18 hours. As internal loading control, anti-β-actin primary antibody was used. Clones, sources and dilutions of the primary antibodies used herein are summarized in Table 3. After washing with PBS, the membranes were incubated with species specific horseradish peroxidase-conjugated secondary antibodies (listed in Table 3) for 60 min at RT. Immunoblots were visualized by home made "enhanced chemiluminescence" ECL [39]. Resulting images were quantified using ImageJ software (NIH, Bethesda, USA).

**Table 3:** Antibodies for Western blot and immunohistochemistry

Gene	Application	Protein forms detected by WB	Species	Type/clone	Dilution In WB	Dilution in IHC-P and ICC	Company
MMP-1	WB, IHC	latent and active	rabbit	polyclonal	1:750	1:100	Biozol

MMP-2	WB, IHC	latent and active	rabbit	polyclonal	1: 1000	1:100	Abcam

MMP-3	WB, IHC, ICC	latent and active	mouse	SPM 293	1: 500	1:50	Abcam

MMP-7	WB	latent and active	mouse	111433	1: 500	-	Abcam

MMP-8	WB	latent and active	mouse	115-13D2	1: 1000	-	Chemicon

MMP-9	WB	latent and active	mouse	9D4.2	1: 500	-	Chemicon

MMP-10	WB, IHC, ICC	latent and active	mouse	IVC5	1: 500	1:100	Chemicon

MMP-11	WB	latent and active	mouse	SL 3.01	1: 500	-	Abcam

MMP-11	IHC, ICC	latent and active	mouse	SPM 199	-	Prediluted	Biozol

MMP-12	WB	latent and active	rabbit	polyclonal	1: 1000	-	Abcam

MMP-13	WB, IHC	latent and active	mouse	87512	1: 500	1:100	R&D

MMP-14	WB, IHC, ICC	latent and active	rabbit	polyclonal	1: 500	1:75	Abcam

MMP-15	WB, IHC, ICC	latent and active	rabbit	polyclonal	1: 500	1:100	Abcam

MMP-19	WB	latent and active	rabbit	polyclonal	1: 3000	-	Biozol

MMP-19	IHC, ICC	latent and active	rabbit	polyclonal	-	Prediluted	Biozol

MMP-23	WB	latent and active	rabbit	polyclonal	1: 1000	-	Abcam

MMP-24	WB	latent and active	rabbit	polyclonal	1: 1000	-	Abcam

MMP-27	WB	not specified	rabbit	polyclonal	1: 1000	-	Abcam

MMP-28	WB	not specified	rabbit	polyclonal	1: 1000	-	Abcam

β-actin	WB	b-actin	mouse	M/Abcam 8226	1: 10.000	-	Abcam

WB: Western blot; IHC-P: Immunohistochemistry (formalin-fixed paraffin-embedded section),

ICC: Immunocytochemistry

### Immunohistochemistry

For immunohistochemistry, tissue samples were cut at 2 μm from formalin-fixed, paraffin-embedded tissue blocks, placed on adhesive treated slides (Superfrost, Langenbrinck, Emmendingen, Germany) and dried overnight at room temperature. Paraffin sections were dewaxed twice with xylene and rehydrated in a graded series of ethanol and in distilled water. The sections were stained without pretreatment for antigen demasking. Only in the case of MMP-19, slides were pretreated in the microwave oven in a 10 mM sodium citrate buffer solution (pH 6.0) for 10 minutes (750 W/s). Endogenous peroxidase activity was blocked with 0.3% hydrogen peroxide in methanol for 10 minutes. To reduce non-specific binding capacity of the tissue, slides were treated with a solution of human immunoglobulin (Beriglobin; Aventis Behring, Marburg, Germany) in phosphate buffered saline (PBS, dilution 1:50) for 15 minutes at room temperature. Afterwards the sections were incubated overnight at 4°C with one of the respective primary antibodies against MMP-1, -2, -3, -10, -11, -13, -14, -15 and -19 diluted in antibody diluent (DAKO, Hamburg, Germany; antibodies are listed in Table 3). The other antibodies listed in Table 2 did not produce sufficient staining results for immunohistochemistry and could therefore not be analyzed herein. After washing with PBS, the sections were incubated with horseradish-peroxidase (HRP)-labeled LSAB2 kit (streptavidin-biotin system; DAKO). Peroxidase activity was developed with diaminobenzidine (DAB; DCS, Hamburg, Germany) as a substrate for 5 min, which resulted in brown staining. The slides were counterstained with haematoxylin, dehydrated in graded ethanol, embedded in Vitro Clud (Langenbrinck) and analyzed using a light microscope Othoplan (Leica, Germany).

### Immunocytochemistry

For immunocytochemistry, cells harvested with accutase were diluted at 1 × 10^6 ^cells/ml. 20 μl of each cell line was applied on APES-treated slides, air dried and fixed in a 2% formalin solution (diluted in PBS) for 15 min. Cells were then permeabilized by incubation in 0.05% Triton X100 in PBS for 10 min followed by a single wash in PBS. Blocking of non-specific binding capacities, incubation and detection of primary antibodies as well as counterstaining and embedding was performed as described for immunohistochemistry. All incubation steps apart from the primary antibodies were carried out at room temperature.

Antibodies were validated for this staining technique using cell lines of different cancer sources (breast, cervix, placenta, ovary and endometrium) with known MMP expression in RT-PCR and/or WesternBlot. With the staining protocol applied, only MMP-3, -11, -14, -15 and -19 produced reproducible results.

### Data analysis and statistics

The intensity of GelRed luminescence and protein expression in Western Blot images was quantified densitometrically using ImageJ software (NIH, Bethesda, USA) and normalized in respect to the corresponding fragment concentration of the ubiquitously expressed genes PBGD and β-actin. Four different expression levels were considered in respect of their densitometrical value. Value 0 was considered to be no expression. Values between 1 and 19 were considered as very weak ((+)), between 20 and 49 as weak (+), between 50 and 79 as moderate (++) and between 80 and 100 as high (+++) expression.

Box-plots were generated using GraphPad Prism 4.0 Software (GraphPad Software, La Jolla, USA). Comparison of expression values between the groups was performed by the non-parametric two-tailed Mann-Whitney-U-test and P-values < 0.05 were considered as statistically significant.

## Results

### Patients' data

We analyzed ten tissue samples of both, grade 2 (G2) and grade 3 (G3) breast cancer tissues. Altogether, patients with grade 3 tumors had a bigger tumor size (T). Furthermore, more patients with G3 tumors had lymph node metastases at the time of the initial diagnosis (7 in G3 vs. 3 in G2 group) or lymphangiosis (8 patients in the G3 vs. 2 patients in the G2 group; Table 1a). Patients of the G2-tumor group were younger than patients in the G3-tumor group (64 vs. 71 years) at initial diagnosis. Concerning tumor type and receptor-status no differences between the two groups were found (Table 1).

### Expression of MMP mRNA in normal breast tissue and breast cancer tissue

**Figure 1:**
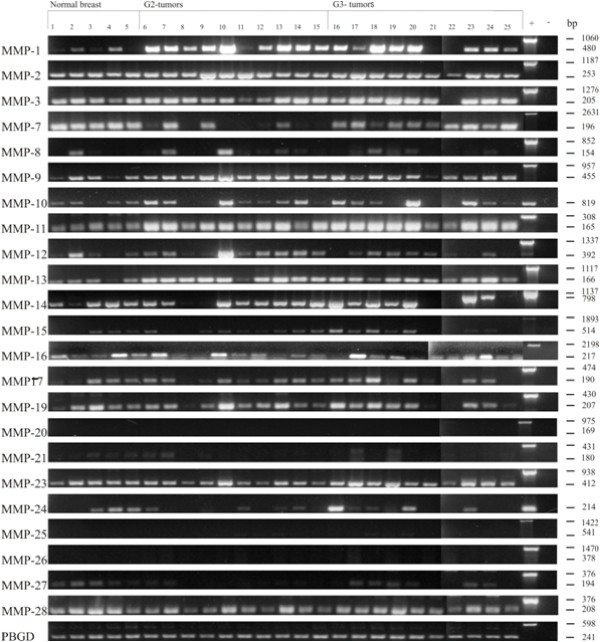
**Expression of MMP mRNA in human normal breast and breast cancer tissue analyzed by semiquantitative RT-PCR**. Total mRNA from normal breast, breast cancer tissue grade 2 (G2) and breast cancer tissue grade 3 (G3) samples was used as template for RT-PCR analysis. Primers used in PCR reactions were designed in flanking exons, specific for each MMP transcripts (primer sequences are listed in Additional file 2). The gene porphobilinogen deaminase (PBGD) was used as internal loading control and the amount of each cDNA was normalized to the amount of PBGD. Genomic DNA amplified by the primer pair as well served as positive control (+).

**Figure 2:**
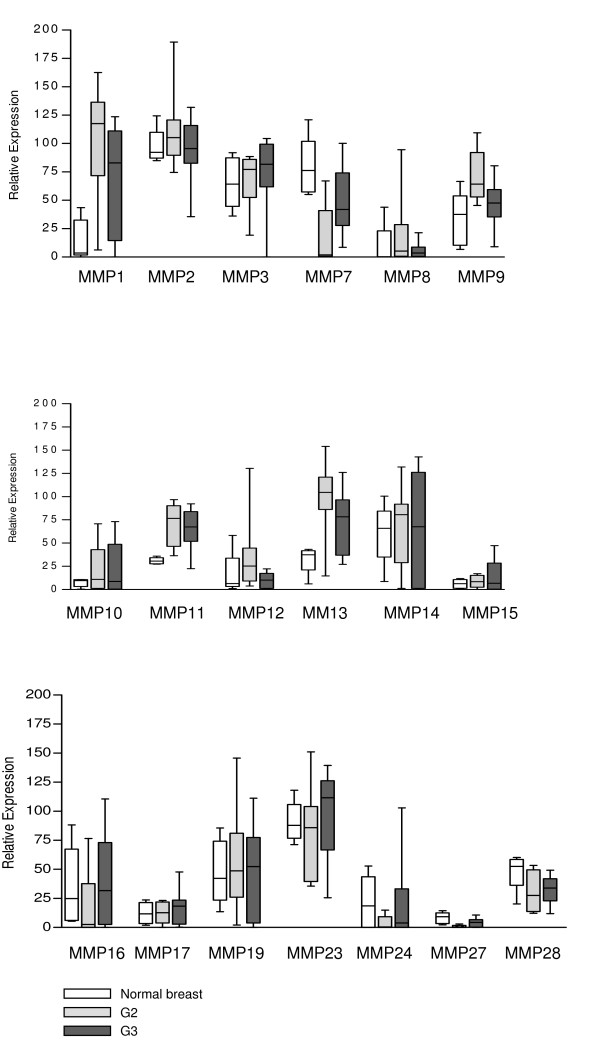
**Boxplot analysis of densitometrically quantified expression of MMP mRNA**. The expression level of each gene was normalized to the corresponding expression of PBGD. For each MMP three boxes are mapped: expression in normal breast tissue (white box), in grade 2 tumors (G2) (light grey box) and in grade 3 tumors (G3) (dark grey box). Black line within the box represents the median, boxes show the quartiles and bars indicate the minimum and maximum value.

Results of the semiquantitative RT-PCR are summarized in Figure 1 and boxplot analysis of densitometrically quantified expression of MMPs in Figure 2. P-values are listed in Table 4. Except for MMP-20 and -26, the mRNA of all other MMPs was detected in breast cancer tissue as well as in normal breast tissue. In summary, three different mRNA expression patterns could be observed comparing normal and malign breast tissue.

**Table 4:** P-values obtained by comparison of the MMP-mRNA expression between normal breast tissue and breast cancer tissue grade 2 and grade 3 respectively (Man-Whitney-U-Test)

	NB-G2	NB-G3	G2-G3
MMP-1	p < 0,01	ns	ns

MMP-2	ns	ns	ns

MMP-3	ns	ns	ns

MMP-7	p < 0,01	p < 0,05	p < 0,05

MMP-8	ns	ns	ns

MMP-9	p < 0,05	p < 0,05	p < 0,05

MMP-10	ns	ns	ns

MMP-11	p < 0,001	p < 0,05	ns

MMP-12	ns	ns	p < 0,05

MMP-13	p < 0,01	ns	ns

MMP-14	ns	ns	ns

MMP-15	ns	ns	ns

MMP-16	ns	ns	ns

MMP-17	ns	ns	ns

MMP-19	ns	ns	ns

MMP-23	ns	ns	ns

MMP-24	ns	ns	ns

MMP-27	ns	ns	ns

MMP-28	ns	p < 0,05	ns

ns = not significant

#### MMPs with higher expression in breast cancer tissue

Significantly higher mRNA expression in breast cancer tissue in contrast to normal breast tissue was detected for MMP-1, -9, -11, -13 and -28 (Figure 1, Table 4). Expression of MMP-1 mRNA was higher in breast cancer tissue grade 2 than in normal breast tissue (p = 0,0027) (Figure 2, Table 4). Similar significant differences between normal breast tissue and grade 2 breast cancer tissue concerning the mRNA levels were observed for MMP-9 (p = 0,0127), MMP-11 (p = 0,0007) and MMP-13 (p = 0,008) (Figure 2). Furthermore MMP-9, -11 and -28 mRNA expression was higher in breast cancer tissue grade 3 than in normal breast tissue (Figure 1 and 2, Table 4). MMP-1 and -13 also showed a higher expression of mRNA in grade 3 breast cancer tissue compared to normal breast tissue, but these differences were not statistically significant. Except for MMP-9 and -12 there was no statistically significant difference in the mRNA expression profile of the remaining three MMPs between G2 and G3 breast cancer tissues. For MMP-9, a higher amount of mRNA was detected in grade 2 breast cancer tissue when compared to grade 3 breast cancer tissue (p = 0,023) (Figure 2).

#### MMPs with lower expression in breast cancer tissue

The expression level of MMP-7 mRNA was higher in normal breast tissue than in breast cancer tissue, both in grade 2 (p = 0,008) and grade 3 (p = 0,04) (Figure 2, Table 4). Interestingly, the expression level of MMP-7 mRNA was lower in grade 2 compared to grade 3 tumors (p = 0,0115) (Figure 2, Table 4).

#### MMPs with equal expression in normal breast tissue and breast cancer tissue

In contrast to the abovementioned MMPs, an equal expression of mRNA was detected in normal breast and breast cancer tissue for MMPs -2, -3, -8, -10, -12, -14, -15, -16, -17, -19, -23, -24 and -27 (Figure 1 and 2). However, for MMP-8, -10, -12, -14 and -24 a diverse expression profile was identified in the tissue samples analyzed. In some tumor samples, a very strong expression was detected, whereas in others no expression could be identified. In addition, the expression level of MMP-21 and -25 was very low in normal and tumor tissues. Therefore, their expression was not quantified densitometrically.

MMPs, which showed obvious changes in their expression in breast cancer tissue in comparison to normal breast tissue could have an influence on breast cancer development. Therefore we analyzed them on protein level further.

### Expression of MMP protein in normal breast tissue and breast cancer tissue

**Figure 3:**
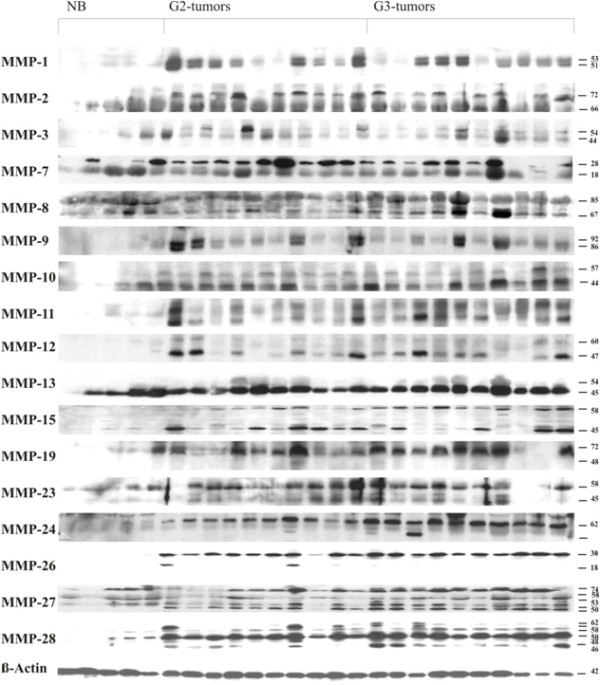
**Expression of MMP protein in human normal breast and breast cancer tissue analyzed by Western blot**. Protein lysates were isolated from normal breast, breast cancer tissue grade 2 (G2) and breast cancer tissue grade 3 (G3). Protein samples were separated by polyacrylamide gel electrophoresis and expression of MMP proteins was visualized using specific antibodies (listed in Table 3). β-actin was used as internal loading control. Inactive (latent) and active forms of MMP proteins are indicated with arrows.

**Figure 4:**
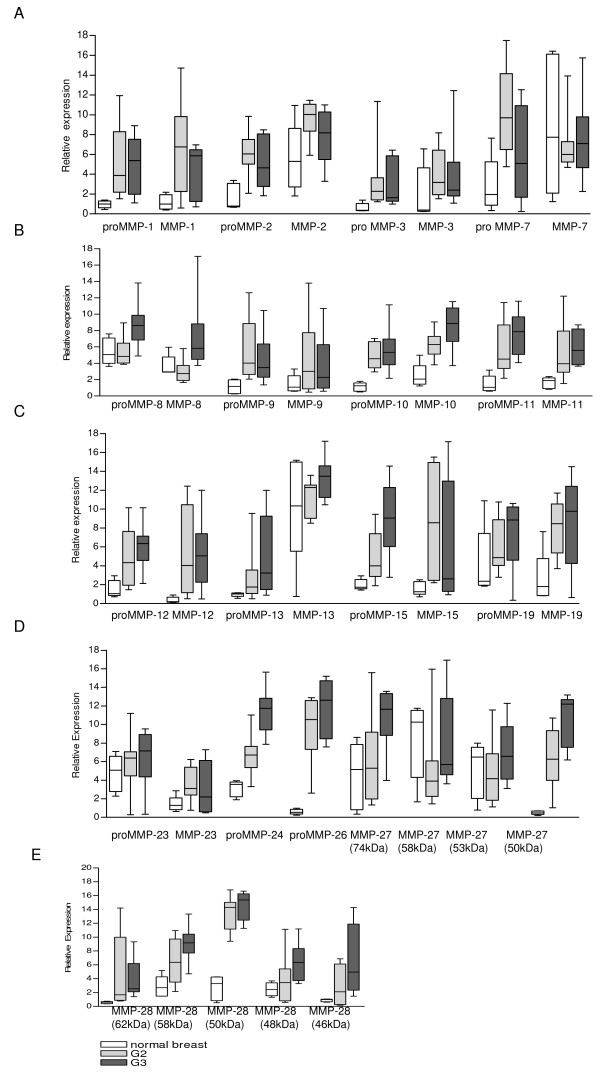
**Boxplot analysis of densitometrically quantified expression of MMP protein**. Protein levels were normalized to the corresponding expression of β-actin. For each MMP six boxes are mapped. The first three boxes are representing the expression of the inactive MMP form (white box: expression in normal breast tissue, light grey box: expression in grade 2 (G2) and dark grey box: expression in grade 3 (G3) breast cancer tissue). The remaining three boxes represent the expression of the active MMPs. The line within the boxplot corresponds to the median value, the box length to the interquartile range, and bars indicate the smallest and largest observations.

To confirm the results obtained by RT-PCR on protein level, proteins were isolated from the same tissue samples, which were used for the mRNA extraction. Using specific antibodies against MMPs (listed in Table 3) a distinction between the latent pro-form and the active form of the MMPs was possible. Results of Western blots are summarized in Figure 3 and boxplot analyses of the densitometrically quantified MMP proteins are shown in Figure 4. Corresponding p-values are listed in Table 5.

**Table 5:** P-values obtained by comparison of the MMP-protein expression between normal breast tissue and breast cancer tissue grade 2 and grade 3 respectively (Man-Whitney-U-Test)

	NB-G2	NB-G3	G2-G3
ProMMP-1	p < 0,001	p < 0,01	ns
MMP-1	p < 0,05	p < 0,05	ns

proMMP-2	p < 0,01	p < 0,01	ns
MMP-2	ns	ns	ns

proMMP-3	p < 0,01	p < 0,01	ns
MMP-3	ns	ns	ns

proMMP-7	p < 0,01	ns	ns
MMP-7	ns	ns	ns

proMMP-8	ns	p < 0,05	p < 0,01
MMP-8	ns	p < 0,05	p < 0,001

proMMP-9	p < 0,001	p < 0,01	ns
MMP-9	ns	ns	ns

proMMP-10	p < 0,001	p < 0,001	ns
MMP-10	p < 0,01	p < 0,01	p < 0,05

proMMP-11	p < 0,01	p < 0,001	ns
MMP-11	p < 0,01	p < 0,001	ns

proMMP-12	p < 0,05	p < 0,01	ns
MMP-12	p < 0,01	p < 0,01	ns

proMMP-13	ns	p < 0,01	ns
MMP-13	ns	ns	ns

proMMP-15	p < 0,01	p < 0,01	ns
MMP-15	p < 0,01	ns	ns

proMMP-19	ns	ns	ns
MMP-19	p < 0,01	ns	ns

proMMP-23	ns	ns	ns
MMP-23	p < 0,05	ns	ns

proMMP-24	p < 0,01	p < 0,001	ns

proMMP-26	p < 0,001	p < 0,001	ns

MMP-27 (74 kDa)	ns	p < 0,01	p < 0,05
MMP-27 (58 kDa)	ns	ns	p < 0,05
MMP-27 (53 kDa)	ns	ns	ns
MMP-27 (50 kDa)	p < 0,001	p < 0,001	p < 0,01

MMP-28 (62 kDa)	p < 0,001	p < 0,001	ns
MMP-28 (58 kDa)	p < 0,05	p < 0,01	ns
MMP-28 (50 kDa)	p < 0,001	p < 0,001	ns
MMP-28 (48 kDa)	ns	p < 0,01	ns
MMP-28 (46 kDa)	ns	p < 0,001	ns

ns = not significant

For MMP-1, -8, -10, -11, -12 and -15 both, expression of proMMP and active MMP were significantly higher in breast cancer tissue compared to normal breast tissue (e.g. expression of proMMP-1 was higher in breast cancer tissue grade 2 (p = 0,0007) and grade 3 (p = 0,0027) when compared to normal breast tissue; Figure 4). All MMPs (except proMMP-19 and proMMP-23 analyzed showed a significantly higher expression of their latent form in breast cancer tissue in comparison to normal breast tissue. Furthermore, for MMP-19 and -23 a significantly higher expression of the active protein could be observed in breast cancer tissue grade 2 when compared to normal breast tissue (Figure 4, Table 5). For MMP-27, four bands at approximately 74, 58, 53 and 50 kDa could be observed (Figure 3). For MMP-28 five bands at approximately 62, 58, 50, 48 and 46 kDa were detected and all of them showed a significantly higher expression in breast cancer tissue, either grade 2 or grade 3, when compared to normal breast tissue (Figure 4).

### Immunostaining of tissue samples

**Figure 5:**
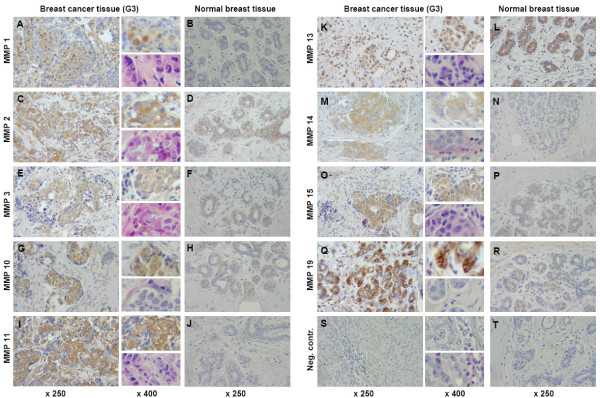
**Immunostaining of MMP expression on paraffin embedded breast cancer tissue sections**. MMP-1, -2, -3, -10, -11, -13, -14, -15 and -19 expression (brown colour) in normal breast tissue and breast cancer tissue grade 3 (G3) was visualized using specific antibodies (listed in Table 3). Staining was predominantly restricted to the cytoplasm and nuclei of tumor cells, whereas staining of stromal cells was rare, e.g. immunostaining of MMP-1, -2 and -10 shows a clear preference for the tumor cell nuclei with slight to moderate additional staining of the cytoplasm of tumor cells (A, C, G). MMP-13 showed a weak to moderate staining of the tumor cells cytoplasm as well as a staining of the cytoplasm of normal breast cells (K and L). MMP-15 showed a strong staining in the cytoplasm and in some nuclei of tumor cells and additional slight staining of stromal cells (O). MMP-11 and -19 staining was strong in the cytoplasm of tumor cells with additional slight staining of stromal cells and some nuclei of tumor cells (I and Q). MMP-3 and -14 showed a clear predominant staining of the tumor cells cytoplasm (E and M). Immunostaining of normal breast tissue served as control. Except MMP-13 (L), low to no staining for the abovementioned MMPs was found in normal breast tissue. Tissue stained with an irregular antibody served as negative control (S and T). Magnification: ×250 for all pictures, ×400 for detail included into the upper right corner adjacent to the corresponding tumor tissue picture; ×400 for HE-staining included into the bottom right corner adjacent to the corresponding tumor tissue picture; brown: DAB, blue: hematoxylin counter stain, purple: hematoxylin-eosin counter stain.

To identify the specific localization of cells expressing MMPs within the tumor sample, we performed immunohistochemistry on paraffin embedded sections and breast cancer tissue grade 3 that were directly adjacent to the sections used for RT-PCR and Western blot. Based on the results of Western blots, we performed immunostaining with antibodies against those MMPs that demonstrated a specific expression pattern related to breast cancer development. Immunostaining with MMP-1, -2, -3, -10, -11, -13, -14, -15 and -19 antibodies corroborates the findings of the Western blot analysis. Typical immunostaining results are shown in Figure 5.

To demonstrate that higher amounts of MMP protein in Western blot analysis were not only caused by the higher proportion of epithelial cells in tumor tissue in comparison to normal breast tissue we performed immunostaining of normal breast tissue sections as well.

For MMP-1 the staining showed a clear predominance for the nuclei of tumor cells with a slight additional staining in the tumor cell's cytoplasm. A weak staining was found in the surrounding stromal/endothelial and immune cells (Figure 5A). In contrast to this no staining of MMP-1 was found in normal breast tissue. Nuclei of tumor cells were also stained with MMP2. However, herein the cytoplasm of tumor cells showed a strong signal and even several stromal cells were found to be clearly positive (Figure 5C). MMP-2 was also found in the cytoplasm of normal breast endothelial cells, albeit not in their nuclei (Figure 5D). Immunostaining of MMP-3 showed a slight staining of the tumor cell's cytoplasm with no staining of the surrounding stromal and immune cells (Figure 5E). The cytoplasm of normal breast endothelial cells showed only a slight positivity for MMP-3. For MMP-10, nuclei and cytoplasm of tumor cells were positive, whereas no staining was found for stromal and immune cells (Figure 5G). The cytoplasm of tumor cells expressed a high amount of MMP-11 with several adjacent stromal cells being positive, too (Figure 5I). As opposed to this, expression of MMP-11 could not be detected in normal breast tissue. For MMP-13 a weak to moderate staining of the cytoplasm and nuclei of tumor cells and, in addition of the cytoplasm of normal breast cells was found (Figure 5K and 5L). Staining of MMP-14 was clearly restricted to the cytoplasm of tumor cells and only very weak in stromal cells (Figure 5M). Again, no MMP-14 could be detected in normal epithelial breast cells. MMP-15 showed a strong staining in the cytoplasm of tumor cells with additional slightly staining of few stromal cells and in some nuclei of tumor cells (Figure 5O). A clear predominant staining of tumor cell's cytoplasm was found for MMP-19 (Figure 5Q). In normal epithelial breast cells only a slight staining of the cytoplasm could be found for MMP-15 and -19.

### Expression of MMP mRNA and protein in breast cancer cell lines

**Figure 6:**
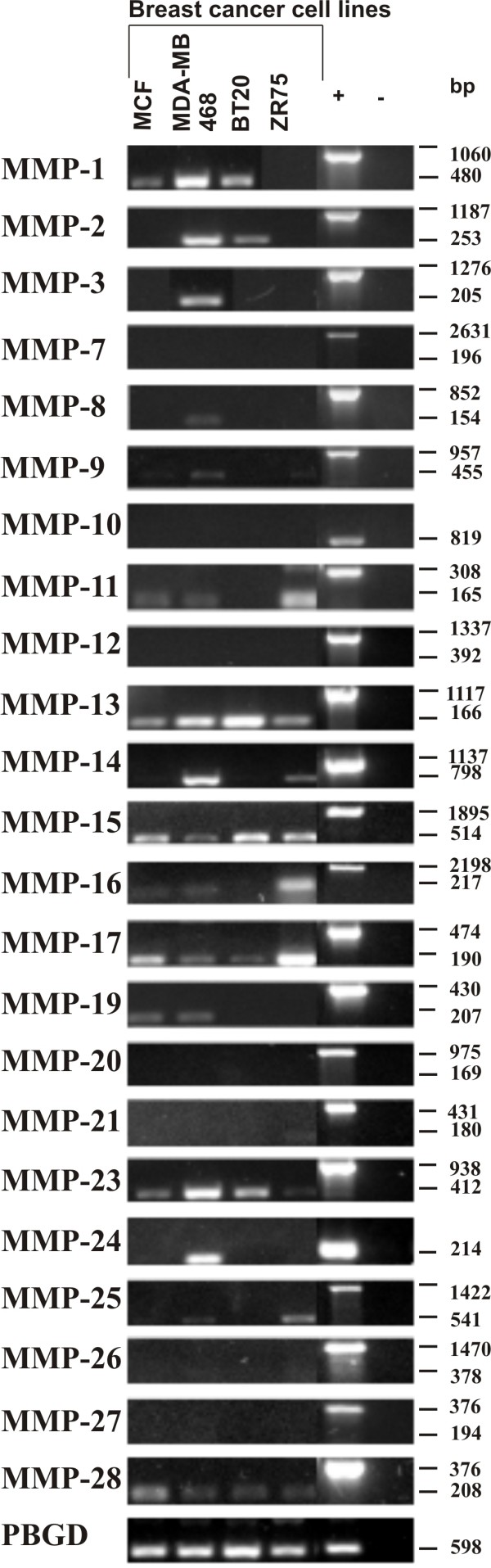
**Expression of MMPs in different human breast cancer cell lines analyzed by semiquantitative RT-PCR**. Total RNA from breast cancer cell lines MCF-7, MDA-MB-468, BT 20 and ZR 75/1 was extracted and used as template for RT-PCR analysis. Primers, specific for each transcript, were designed in flanking exons (see Additional file 2: primer sequences), resulting in longer amplicons if human genomic DNA was amplified (positive control (+)) and in shorter amplicons representing cDNAs. The gene PBGD was used as internal loading control and amounts of cDNA were normalized to the amount of PBGD for each sample.

**Figure 7:**
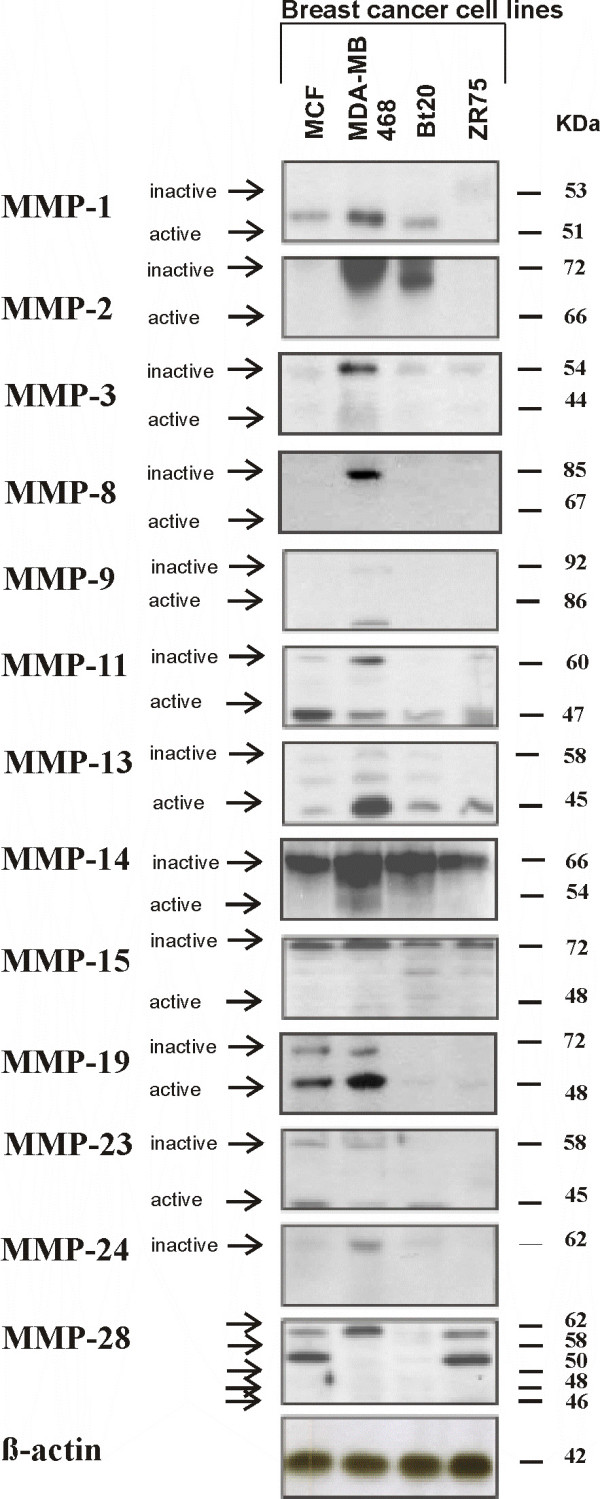
**Expression of MMPs in different human breast cancer cell lines analyzed by Western blot**. Protein lysates were isolated from the four breast cancer cell lines and separated by polyacrylamid gel electrophoresis. MMP proteins were visualized using specific antibodies, capable of recognizing both, the inactive and active forms of MMPs (arrows inactive/active). β-actin was used as an internal loading control. Protein amounts were quantified using the Bradford method and normalized to the amount of β-actin for each sample.

The expression of all human MMPs, which are identified till now, was analyzed in four cell lines: MCF-7, MDA-MB-468, BT 20 and ZR 75/1. Semiquantitative RT-PCR and Western blot results are summarized in Figure 6 and Figure 7 and analyses of densitometrically quantified expression of MMP mRNAs and proteins are shown in Table 6 and Table 7, respectively.

**Table 6:** Relative expression of MMP mRNA in different breast cancer cell lines

	MCF-7	MDA-MB 468	BT 20	ZR 75/1
MMP-1	+	+++	++	0

MMP-2	0	+++	++	0

MMP-3	0	+++	0	0

MMP-7	0	0	0	0

MMP-8	0	(+)	0	0

MMP-9	(+)	(+)	0	(+)

MMP-10	0	0	0	0

MMP-11	+	+	0	+++

MMP-12	0	0	0	0

MMP-13	+	++	+++	++

MMP-14	(+)	+++	(+)	++

MMP-15	++	+	++	++

MMP-16	+	+	0	++

MMP-17	++	+	+	+++

MMP-19	++	++	0	0

MMP-20	0	0	0	0

MMP-21	0	0	0	(+)

MMP-23	++	+++	+++	+

MMP-24	0	++	0	0

MMP-25	0	(+)	0	+

MMP-26	0	0	0	0

MMP-27	0	0	0	0

MMP-28	++	+	+	+

0 = no expression, (+) = very weak expression, + = weak expression, ++ = moderate expression, +++ = high expression

**Table 7:** Relative expression of MMP protein in different breast cancer cell lines

	MCF-7	MDA-MB 468	BT 20	ZR 75/1
proMMP-1	0	0	0	0
MMP-1	+	++	+	0

proMMP-2	0	+++	+++	0
MMP-2	0	0	0	0

proMMP-3	0	++	0	0
MMP-3	0	(+)	0	0

proMMP-8	0	++	0	0
MMP-8	0	0	0	0

proMMP-9	0	(+)	0	0
MMP-9	0	+	0	0

proMMP-11	(+)	+	0	(+)
MMP-11	++	+	(+)	+

proMMP-13	0	(+)	(+)	0
MMP-13	(+)	+++	+	+

proMMP-14	(+)	(+)	(+)	(+)
MMP-14	++	++	++	+

proMMP-15	++	++	+	+
MMP-15	0	(+)	(+)	0

proMMP-19	+	+	0	0
MMP-19	++	+++	0	0

proMMP-23	+	+	0	0
MMP-23	++	(+)	+	0

proMMP-24	(+)	+	(+)	(+)
MMP-24	0	0	0	0

MMP-28(62)	+	++	(+)	+
MMP-28(58)	+++	0	0	+++
MMP-28(50)	0	0	0	0
MMP-28(48)	0	0	0	0
MMP-28(46)	0	0	0	0

0 = no expression, (+) = very weak expression, + = weak expression, ++ = moderate expression, +++ = high expression.

For MMP-13, -14, -15, -17, -23 and -28, mRNA expression was detected in all four cell lines investigated. By Western blot, inactive and active forms of MMP-13, -23 and -28 could be detected to different amounts, whereas for MMP-14 and 15 only the zymogens could be identified in all four cell lines. mRNA of MMP-3, -8 and -24 was identified in the MDA-MB-468 cell line only. Whereas on protein level only the inactive form of these three MMPs was detected. In this cell line, the inactive form of MMP-24 was also identified by Western blot. Only BT-20 was negative for MMP-9 by RT-PCR. However, on protein level, the pro- and active forms of MMP-9 were detected in the MDA-MB-468 cell line only. For MMP-1, strong mRNA expression was detected in the MDA-MB-468 cells and moderate to weak expression in MCF-7 and BT 20. Corresponding to this, on protein level, the active form of MMP-1 could be detected in all three cell lines. The strong MMP-2 mRNA expression correlated with a strong expression of proMMP-2 in the MDA-MB-468 and BT 20 cell lines, whereas the active form of MMP-2 could not be detected in any of the breast cancer cell lines tested. For MMP-11 both, inactive and active forms could be detected in all samples analyzed. Diverse expression patterns were detected for the mRNA of MMP-16, -19 and -25. A weak expression of proMMP-19 and a normal to strong expression of active MMP-19 was detected in the MDA-MB-468 and MCF-7 cell lines. For the remaining MMPs, no expression could be detected in all four breast cancer cell lines by semiquantitative RT-PCR analysis.

### Immunocytochemistry of cell lines

In order to confirm the obtained results of the Western blot analysis we performed an immunocytochemical analysis of MMP-1, -2, -3, -11, -13, -14, -15 and -19 on MDA-MB-468 cells. For these MMPs, MDA-MB-468 cells showed the strongest protein expression using Western Blot analysis and therefore seemed to be the most promising cell line for the immunocytochemical analysis.

**Figure 8:**
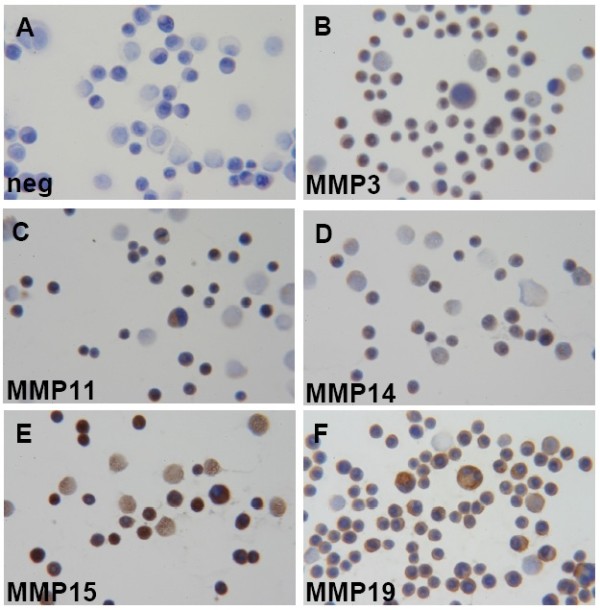
**Expression of MMPs in MDA-MB-468 cells analyzed by immunocytochemistry**. MMP-3, -11, -14, -15 and -19 expression (brown colour) in MDA-MB-468 cells was visualized using specific antibodies (listed in Table 3). Cells stained with a non-specific primary antibody served as negative control (A). Magnification: ×400 for all pictures; brown: DAB, blue: hematoxylin counter stain.

Albeit MMP-1, -2 and -13 did not stain with either the MDA-MB cell line or with control cells, immunocytochemistry of the MDA-MB-468 cell line corroborates the results obtained by Western blot analysis. Results are shown in Figure 8.

Weak cytoplasmatic positivity was seen in all vital cells for MMP-3, which in addition was found in the majority of the nuclei, too (Figure 8B). A moderate to strong expression of MMP-11 and -14 was identified in nearly all MDA-MB-468 cells (Figures 8C and 8D). MMP-15 was highly expressed in all cells (Figure 8E) and MMP-19 showed a clear positivity in all cells, too (Figure 8F). In both cases, MMPs were localized in the cytoplasm.

## Discussion

Breast cancer is an epithelial tumor with high invasive and metastatic potential. Poorly differentiated tumors (G3) seem to have a higher invasive potential resulting in a higher frequency of lymph node metastases and lymphangiosis carcinomatosa than well differentiated tumours (G1) [40]. These aspects were also found in the G3 tumor patient group investigated in this study (Table 1).

In our study we focused on the expression analysis of human specific matrix metalloproteinases (MMPs) in breast cancer specimens. The expression analysis showed three different expression patterns: high, low or equal expression of MMPs in breast cancer tissue compared to normal breast tissue. For the three collagenases, MMP-1, -8 and -13, the first type of the expression pattern was identified. Our data about MMP-1 are in correlation with previously published results [5,18,20,41]. We found the increase of the expression of latent and active protein forms of MMP-1 to be related to higher tumour grade. This finding fits with previous published data, reporting on a shortened relapse-free-survival in breast cancer patients with high expression of MMP-1 [15]. Published data about the site of the MMP-1 production are contradictory [42]. However, we showed that MMP-1 expression is clearly restricted to the tumour cells, thus confirming the findings of Iwata et al. [41]. For MMP-8, some studies indicate its inhibitory effect on building metastatic formations [17,43], and no correlation between MMP-8 expression in breast cancer and negative prognostic factors was found using ELISA [44]. In contrast to this, we found a significant higher expression of pro- and active MMP-8 in breast cancer compared to normal breast tissue. A correlation between the high expression of MMP-13 in breast cancer tissue and a higher rate of distant metastases, poor prognosis and in addition a higher expression of MMP-13 in early stage tumors was shown [15,45]. In accordance to this, we also identified higher amounts of MMP-13 mRNA and pro-form in breast cancer tissue. Similar to the findings of Zhang and colleagues [45], we identified MMP-13 protein to be predominately expressed in the cytoplasm of tumor cells. The expression of the two gelatinases, MMP-2 and MMP-9, in breast cancer is well investigated in many studies using different methods [13,16,19,20,41,46]. Our Western blot data showed a significant increase of proMMP-2 expression in tumor tissue, which is in accordance with data described [15,19,46]. In addition, MMP-2 staining was specific for nuclei and cytoplasm of tumor cells. This is in accordance with other studies, verifying MMP-2 to be an unfavorable prognostic factor in breast cancer [5,13]. Our findings about the high expression of MMP-9 in the specimens analyzed are in accordance with data published by Przybylowska and colleagues, who found increased levels of MMP-9 to correlate with G3 breast cancer [20]. In contrast, studies of Jones and Rhako failed to demonstrate any association between the expression of MMP-9 in carcinoma or stromal cells and clinicopathological parameters using immunohistochemistry [47,48]. These differences might be due to different detection methods used. For the matrilysin MMP-7 only few contradictory data are available suggesting that its role in breast cancer has not been brightly investigated yet [15,16,19,42]. We also found divergent data concerning the expression of MMP-7 in our tissue samples. While the expression of MMP-7 mRNA was significantly lower in breast cancer tissue when compared to normal breast tissue. Equal to higher expression levels of MMP-7 pro and active forms in breast cancer tissue were detected. This could be due to a regulation on the translational level, resulting in low levels of MMP-7 mRNA in breast cancer tissue and – as effect of a higher transcriptional rate in breast cancer tissue – higher protein levels of MMP-7 in breast cancer tissue. In accordance to this, the expression of the latent form of MMP-7 was stronger than the expression of its active form in the analyzed samples, like in the case of MMP-23 and -24, as mentioned above. Although the amount of MMP-10 (stromelysin 2) on mRNA level was equal in all analyzed samples, expression of its pro and active protein form was significantly higher in breast cancer samples.

Using immunohistochemistry we found a nuclear staining for MMP-1, -2 and -10, which is unusual, because MMPs are considered to be cytoplasmatic or membrane-bound proteins. The nuclear localization of the abovementioned MMPs could thus be an indication of further new functional roles of MMPs. In accordance to this, an atypical nuclear staining was also observed by Ip et al. for MMP-14 in hepatocellular carcinoma [49]. In this immunohistochemical study the nuclear localization of MMP-14 was associated with aggressive tumor features including poor prognosis.

In accordance to our data, that clearly show a significant increase in the expression of MMP-11 mRNA, propeptide and peptide in breast cancer compared to normal breast tissue, the studies of Pacheco and Kossakowska reported the same expression pattern for this matrix metalloproteinase [16,19]. For MMP-15 (MT2-MMP) no correlation between its expression, positive axillary node status, distant metastases and size of breast tumor was detected by immunohistochemistry [22]. However, our data show a high expression of its protein in breast cancer tissue for both, latent and active forms. For another membrane bound MMP, MMP-24, only the size of the latent protein was known and its expression was found to be significantly higher in breast cancer tissue. A putative active bandage was detected at about 55 kDa; this could possibly be an active breakdown product of MMP-24, although the precise size of the active form of MMP-24 is not known till now. The expression of its latent form was stronger than the expression of the putative active form in the analyzed samples. The absence of activated MMPs in tumor samples is not unique; this phenomenon was also observed for MMP-9 in ovarian cancer [50]. Expression and activity of proMMP-9, but not its active form, was shown in the aggressive form of this tumor and could reflect the presence of inflammatory cells, which promote tumor progression.

In an immunohistochemical study comparing normal breast tissue and mammary gland tumors, Djonov and colleagues found MMP-19 to be expressed in all benign lesions, whereas no expression was found in tumor tissue [51]. However, we found MMP-19 protein to be higher expressed in cancer tissue although its mRNA was equally expressed in all analyzed samples. These different findings might be due to different antibodies used in both studies. The same expression pattern was identified for the latent and active forms of MMP-12, -27 and -28. Active MMP-23 protein showed also a higher expression in G2 compared to normal breast specimens. However, similar to MMP-24, expression of its active form was weaker than of the latent form. Although the expression of MMP-27 mRNA was lower in breast cancer samples, its protein showed a diverse expression pattern in the analyzed samples.

To date there are very few publications on the most recently described MMP-27 and -28, and only very little information about their protein sizes.

Showing a statistical significant difference in the expression level between normal breast and breast cancer tissue grade 2 and 3 it seems possible, that some of the putative bandages of MMP-27 and -28 are playing a role in breast cancer development. Because till now no precise sizes of their breakdown products and putative active and inactive forms are known, these differences in the expression level between normal breast and breast cancer tissue can give us a first sign that these two MMPs can be involved in tumor progression.

A strong association between the expression of MMP-14 by stromal cells and poor prognosis for the patients was found by Vizoso et al. using immunohistochemistry [15]. However, in our study we found MMP-14 mRNA to be equally expressed in normal breast and breast cancer tissue. These differences might be due to the different expression profiles of MMP-14 on mRNA and protein level. Therefore MMP-14 mRNA could possibly be expressed on equal levels in breast cancer tissue and normal breast tissue. Differences in expression might also be caused on the translational level. Using immunohistochemistry we found MMP-14 protein to be localized in the cytoplasm of tumor cells with only slight additional staining of the surrounding stroma cells, whereas no staining in normal breast tissue could be detected. One possible reason might be a higher translational rate of MMP-14 in breast cancer tissue. MMP14 plays an important role in the activation of other MMPs [52]. For example, MMP-14 activates proMMP-2 at the cell surface [52]. In our study, MMP-2 mRNA was ubiquitously expressed in all analyzed samples, but – however – without a clear correlation to the expression of its activator, MMP-14.

For the remaining MMPs there are only limited or no data available in the literature about their expression in breast cancer tissue. Some studies documented the expression of MMP-3 in breast cancer [5,21,42]. In one of those, significantly higher amounts of proMMP-3 and equal amounts of its active form in breast cancer tissue compared to normal breast tissue were found using zymography [5]. These findings are in accordance with the results obtained in our study. Furthermore, only a weak staining of MMP-3 was found in tumor cells using immunohistochemistry, which thus correlates with published results [5,41]. In contrast to this Haupt et al. found MMP-3 to be located in the stromal tissue that surrounds the epithelial tumor cells [21]. Ueno and colleagues could not detect MMP-16 (MT3-MMP) in breast cancer tissue using Northern blot analysis [22], whereas we detected its mRNA in normal and breast cancer tissue by RT-PCR. These differences in MMP-16 detection could be due to the different sensitivity of the methods used. MMP-17 (MT4-MMP) showed the same expression pattern in all tissues analyzed. For MMP-26 no expression could be found in normal mammary glands, whereas a high expression could be identified in precursor lesions (DCIS) using immunohistochemistry. Further, on tumor progression to invasive carcinomas the expression level of MMP-26 was described to decrease again [53]. This is partly in accordance to our results, as we did not detect MMP-26 mRNA in breast cancer tissue G2 and G3, though we could not detect MMP-26 mRNA in normal breast tissue either. However, PCR products obtained by amplification of the genomic DNA, which was used as positive control, showed that PCR properly worked. As MMP-26 could not be detected by PCR, we did not investigate its expression on protein level. Expression of MMP-21 and MMP-25 was very low on mRNA level, whereas MMP-20 showed no expression. Therefore we did not investigate them in further.

### Expression of MMPs in breast cancer cell lines

Since there is obvious evidence about the influence of MMPs on the development of breast cancer, we studied their expression pattern in four different breast cancer cell lines, which could be used as a model system for the analysis of the regulation of MMP expression in this tumor entity. Our analyses showed, that the MDA-MB-468 cell line expresses sixteen out of twenty- three MMPs analyzed on mRNA and protein level. Using immunocytochemistry, we could confirm our data obtained by RT-PCR and Western Blot analysis in this cell line, as we found a weak staining for MMP-3 in the cytoplasm and nuclei of the tumor cells. A moderate to strong expression of MMP-11 and -14 was identified in nearly all MDA-MB-468 cells, too. MMP-15 and -19 expression was clearly restricted to the cytoplasm of tumor cells using immunocytochemistry.

This is partly in contrast to the results obtained by Gimbernardi et al. [9] and Grant et al. [54], who studied the expression of MMP-1, -2, -3, -7, -8, -9, -10, -11, -12, -13, -14, -15, -16, -17, -18 and -20 by RT-PCR in 84 normal or human cancer cell lines, including some breast cancer cell lines such as BT 20, MCF-7, MDA-MB-468, ZR 75/1 and T47D. They observed the expression of MMP-9, -10, -14 and -15 in MDA-MB-468 cells only, whereas we could detect the expression of MMP-1, -2, -3, -8, -11, -16, -17 and -19 in this cell line too. In contrast to their findings, we did not detect any expression of MMP-7 and MMP-10 on mRNA level. These differences might be due to differences in the sensitivity of the methods and primers used and/or to cell culture conditions. Differences in MMP expression profiles caused by different cell culture conditions were recently shown [55]. Kousidou et al. observed a higher expression of MMP-1 and MMP-11 in MCF-7, BT 20 and ZR 75/1 cell lines when cultured in serum-free media, whereas expression of MMP-9 in these three cell lines was higher when serum was added to the medium [55]. Similar to this, in our serum-cultured cells, we observed a weak to high expression for MMP-1, -2, -13, -15, -17, -23 and -28 in BT 20 cells, whereas Gimbernardi et al. detected MMP-15 only in this cell line. For the remaining MMPs, no expression in BT-20 cells was detected in our study.

## Conclusion

In conclusion we identified MMP-1, -2, -8, -9 -10, -11, -12, -13, -15, -19, -23, -24, -27 and -28 as matrix metalloproteinases which show a stronger expression in breast cancer tissue compared to normal breast tissue and could thus seem to be associated with breast cancer development. Hence, all fourteen MMPs are candidates for future functional analyses and potential targets for new therapeutic agents for patients. Even though first clinical trials using MMP-inhibitors were disappointing [56], this fact highlights the need for a better insight into the mechanisms by which MMPs contribute to tumor invasion and building metastatic formations. This will provide the basis for a better understanding of the role of MMPs in the cellular processes of tumor invasion and building of metastatic formations.

*In vitro *studies can provide a model for further investigations dealing with the role of MMPs. Given the heterogeneity of tumors and their variable growth characteristics, examination of several cell lines of varying properties on the MMP expression is necessary. Our study demonstrates that four analyzed breast cancer cell lines constitutively express a wide variety of MMPs on mRNA and protein level and could be candidates for in vitro model systems studying the role of MMPs in breast cancer biology.

## Competing interests

The authors declare that they have no competing interests.

## Authors' contributions

AK drafted the manuscript, set up the experiments, collected the data, analyzed and interpreted the results. UK participated in the study design, interpretation of the results and finalization of the manuscript. JD participated in editorial support. MK carried out the Western blot analysis and immunohistochemistry. JA participated in the study design, experimental concept, interpretation of the results and drafting of the manuscript. All authors read and approved the final manuscript.

## Pre-publication history

The pre-publication history for this paper can be accessed here:

http://www.biomedcentral.com/1471-2407/9/188/prepub

## Supplementary Material

Additional file 1 **MMP expression in cell lines**. Expression of MMP mRNA and protein in different breast cancer cell lines. Click here for file

Additional file 2 **Primer sequences**. Primers and conditions used for semiquantitative RT-PCR screening. Click here for file
