# Supplementary material for: Expression of matrix metalloproteinases (MMPs) in primary human breast cancer and breast cancer cell lines: New findings and review of the literature
Source: BMC Cancer. 2009 Jun 16;9:188. doi: 10.1186/1471-2407-9-188 (PMC2706257; doi:10.1186/1471-2407-9-188)
Supplement: Additional file 1 — MMP expression in cell lines. Expression of MMP mRNA and protein in different breast cancer cell lines. [file 1471-2407-9-188-S1.doc]

**Additional File 1**: Expression of MMP mRNA and protein in different breast cancer cell lines

|  | **T47D** | | **MDA-MB 231** | | **MDA-MB 435** | | **MCF-7** | | **MDA-MB 468** | | **BT 20** | | **ZR 75/1** | |
| --- | --- | --- | --- | --- | --- | --- | --- | --- | --- | --- | --- | --- | --- | --- |
| **MMP-1** | + | Bartsch et al. [23] | + | Bachmeier et al [24] | + | Bachmeier et al [24] | + | Bachmeier et al [24] | - | Gimbernardi et al [9] | - | Balduyck et al.[27] | - | Gimbernardi et al [9] |
|  | - | Gimbernardi et al [9] | + | Bartsch et al. [23] | + | Haupt et al [26] | + | Bartsch et al. [23] |  |  | - | Gimbernardi et al [9] |  |  |
|  |  |  | + | Stark et al [25] | + | Gimbernardi et al [9] | + | Haupt et al [26] |  |  |  |  |  |  |
|  |  |  | + | Haupt et al [26] | - | Bachmeier et al [24] | - | Bachmeier et al [24] |  |  |  |  |  |  |
|  |  |  | + | Gimbernardi et al [9] |  |  | - | Balduyck et al.[27] |  |  |  |  |  |  |
|  |  |  | - | Haupt et al [26] |  |  |  |  |  |  |  |  |  |  |
| **MMP-2** | - | Bartsch et al. [23] | + | Gimbernardi et al [9] | + | Bachmeier et al [24] | + | Garbett et al.[5] | - | Gimbernardi et al [9] | - | Balduyck et al.[27] | - | Gimbernardi et al [9] |
|  | - | Gimbernardi et al [9] | - | Stark et al [25] | - | Gimbernardi et al [9] | - | Balduyck et al.[27] |  |  | - | Gimbernardi et al [9] |  |  |
|  |  |  |  |  |  |  | - | Bartsch et al. [23] |  |  |  |  |  |  |
|  |  |  |  |  |  |  | - | Garbett et al.[5] |  |  |  |  |  |  |
|  |  |  |  |  |  |  | - | Gimbernardi et al [9] |  |  |  |  |  |  |
| **MMP-3** | - | Bartsch et al. [23] | + | Bachmeier et al [24] | + | Bachmeier et al [24] | + | Bachmeier et al [24] | - | Gimbernardi et al [9] | - | Balduyck et al [27] | - | Gimbernardi et al [9] |
|  | - | Gimbernardi et al [9] | + | Stark et al [25] | + | Haupt et al [26] | + | Haupt et al [26] |  |  |  | Gimbernardi et al [9] |  |  |
|  |  |  | + | Haupt et al [26] | + | Gimbernardi et al [9] | - | Bachmeier et al [24] |  |  |  |  |  |  |
|  |  |  | - | Bartsch et al. [23] |  |  | - | Balduyck et al [27] |  |  |  |  |  |  |
|  |  |  | - | Gimbernardi et al [9] |  |  | - | Bartsch et al [23] |  |  |  |  |  |  |
|  |  |  |  |  |  |  | - | Gimbernardi et al [9] |  |  |  |  |  |  |
| **MMP-7** | + | Bartsch et al. [23] | + | Bachmeier et al [24] | + | Bachmeier et al [24] | - | Bachmeier et al [24] | + | Gimbernardi et al [9] | - | Balduyck et al [27] | + | Gimbernardi et al [9] |
|  | + | Gimbernardi et al [9] | + | Bartsch et al. [23] | + | Gimbernardi et al [9] | - | Bartsch et al. [23] |  |  | - | Gimbernardi et al [9] |  |  |
|  |  |  | + | Gimbernardi et al [9] |  |  | - | Gimbernardi et al [9] |  |  |  |  |  |  |
| **MMP-8** | - | Bartsch et al. [23] | + | Bartsch et al. [23] | - | Gimbernardi et al [9] | - | Balduyck et al.[27] | - | Gimbernardi et al [9] | - | Gimbernardi et al [9] | - | Gimbernardi et al [9] |
|  | - | Gimbernardi et al [9] | - | Stark et al.[25] |  |  | - | Bartsch et al. [23] |  |  |  |  |  |  |
|  |  |  | - | Gimbernardi et al [9] |  |  | - | Gimbernardi et al [9] |  |  |  |  |  |  |
| **MMP-9** | - | Bartsch et al. [23] | + | Bachmeier et al [24] | + | Bachmeier et al [24] | + | Garbett et al.[5] | + | Gimbernardi et al [9] | - | Balduyck et al.[27] | - | Gimbernardi et al [9] |
|  | - | Gimbernardi et al [9] |  | Stark et al.[25] |  | Gimbernardi et al [9] | - | Balduyck et al.[27] |  |  | - | Gimbernardi et al [9] |  |  |
|  |  |  |  | Gimbernardi et al [9] |  |  | - | Bartsch et al. [23] |  |  |  |  |  |  |
|  |  |  |  |  |  |  | - | Garbett et al.[5] |  |  |  |  |  |  |
| **MMP-10** | + | Gimbernardi et al [9] | + | Bachmeier et al [24] | + | Bachmeier et al [24] | + | Bachmeier et al [24] | + | Gimbernardi et al [9] | + | Gimbernardi et al [9] | + | Gimbernardi et al [9] |
|  |  |  | + | Bartsch et al. [23] | + | Gimbernardi et al [9] | - | Bachmeier et al [24] |  |  |  |  |  |  |
|  | - | Bartsch et al. [23] | + | Gimbernardi et al [9] | - | Bachmeier et al [24] | - | Bartsch et al. [23] |  |  |  |  |  |  |
|  |  |  | - | Bachmeier et al [24] |  |  | - | Gimbernardi et al [9] |  |  |  |  |  |  |
| **MMP-11** | + | Bartsch et al. [23] | + | Bachmeier et al [24] | + | Bachmeier et al [24] | + | Bartsch et al. [23] | - | Gimbernardi et al [9] | - | Balduyck et al.[27] | - | Gimbernardi et al [9] |
|  | - | Gimbernardi et al [9] | + | Bartsch et al. [23] | - | Gimbernardi et al [9] | + | Bachmeier et al [24] |  |  | - | Gimbernardi et al [9] |  |  |
|  |  |  | - | Gimbernardi et al [9] |  |  | - | Bachmeier et al [24] |  |  |  |  |  |  |
|  |  |  |  |  |  |  | - | Balduyck et al.[27] |  |  |  |  |  |  |
|  |  |  |  |  |  |  | - | Gimbernardi et al [9] |  |  |  |  |  |  |
| **MMP-12** | - | Bartsch et al. [23] | - | Bartsch et al. [23] | + | Gimbernardi et al [9] | - | Bartsch et al. [23] | - | Gimbernardi et al [9] | - | Gimbernardi et al [9] | - | Gimbernardi et al [17] |
|  | - | Gimbernardi et al [9] | - | Gimbernardi et al [9] |  |  | - | Gimbernardi et al [9] |  |  |  |  |  |  |
| **MMP-13** | + | Bartsch et al. [23] | + | Bartsch et al. [23] | - | Gimbernardi et al [9] | - | Balduyck et al.[27] | - | Gimbernardi et al [9] | - | Balduyck et al.[27] | - | Gimbernardi et al [17] |
|  | - | Gimbernardi et al [9] | + | Stark et al.[25] |  |  | - | Bartsch et al. [23] |  |  |  |  |  |  |
|  |  |  | - | Gimbernardi et al [9] |  |  | - | Gimbernardi et al [9] |  |  |  |  |  |  |
| **MMP-14** | + | Gimbernardi et al [9] | + | Bartsch et al. [23] | + | Bachmeier et al [24] | + | Bartsch et al. [23] | + | Gimbernardi et al [9] | + | Gimbernardi et al [9] | + | Gimbernardi et al [17] |
|  | - | Bartsch et al. [23] | + | Stark et al.[25] | + | Gimbernardi et al [9] | + | Gimbernardi et al [9] |  |  |  |  |  |  |
|  |  |  | + | Haupt et al [26] |  |  | + | Haupt et al [26] |  |  |  |  |  |  |
|  |  |  | + | Gimbernardi et al [9] |  |  |  |  |  |  |  |  |  |  |
| **MMP-15** | + | Bartsch et al. [23] | + | Bartsch et al. [23] | + | Gimbernardi et al [9] | + | Bartsch et al. [23] | + | Gimbernardi et al [9] | + | Gimbernardi et al [9] | + | Gimbernardi et al [9] |
|  | + | Gimbernardi et al [9] | + | Gimbernardi et al [9] |  |  | + | Gimbernardi et al [9] |  |  |  |  |  |  |
| **MMP-16** | + | Bartsch et al. [23] | + | Bartsch et al. [23] | + | Gimbernardi et al [9] | + | Bartsch et al. [23] | - | Gimbernardi et al [9] | - | Gimbernardi et al [9] | + | Gimbernardi et al [9] |
|  | + | Gimbernardi et al [9] | - | Gimbernardi et al [9] |  |  | + | Gimbernardi et al [9] |  |  |  |  |  |  |
| **MMP-17** | + | Gimbernardi et al [9] | - | Gimbernardi et al [9] | + | Gimbernardi et al [9] | + | Gimbernardi et al [9] | - | Gimbernardi et al [9] | - | Gimbernardi et al [9] | + | Gimbernardi et al [9] |
| **MMP-20** | - | Gimbernardi et al [9] | - | Gimbernardi et al [9] | - | Gimbernardi et al [9] | - | Gimbernardi et al [9] | - | Gimbernardi et al [9] | - | Gimbernardi et al [9] | - | Gimbernardi et al [9] |
