# Supplementary material for: Expression of matrix metalloproteinases (MMPs) in primary human breast cancer and breast cancer cell lines: New findings and review of the literature
Source: BMC Cancer. 2009 Jun 16;9:188. doi: 10.1186/1471-2407-9-188 (PMC2706257; doi:10.1186/1471-2407-9-188)
Supplement: Additional file 2 — Primer sequences. Primers and conditions used for semiquantitative RT-PCR screening. [file 1471-2407-9-188-S2.doc]

**Additional File 1** Primers and conditions used for semiquantitative RT-PCR screening

| **Gene** | **Forward primer** | **Reverse primer** | **Tm (°C)** | **Cycles** | **cDNA size (bp)** | **Genomic DNA (bp)** |
| --- | --- | --- | --- | --- | --- | --- |
| MMP-1 | 5’-AAGGCCAGTATGCACAGCTT-3’ | 5’-TGCTTGACCCTCAGAGACCT-3’ | 57 | 32 | 480 | 1.060 |
| MMP-2 | 5’-TTTCCATTCCGCTTCCAGGGCAC-3’ | 5’-TCGCACACCACATCTTTCCGTCACT-3’ | 62 | 32 | 253 | 1.187 |
| MMP-3 | 5’-GGCTTTCCCAAGCAAATAGC -3’ | 5’-GTGCCCATATTGTGCCTTCT -3’ | 57 | 32 | 205 | 1.276 |
| MMP-7 | 5’-TCCAACCTATGGAAATGGAGA-3’ | 5’-GGAGTGGAGGAACAGTGCTT-3’ | 58 | 32 | 196 | 2.631 |
| MMP-8 | 5’-TCTGCAAGGTTATCCCAAGG-3’ | 5’- ACCTGGCTCCATGAATTGTC-3’ | 57 | 32 | 154 | 852 |
| MMP-9 | 5’-CCTGCCAGTTTCCATTCATC-3’ | 5’-GCCATTCACGTCGTCCTTAT-3’ | 58 | 32 | 455 | 957 |
| MMP-10 | 5’-CCAGTCTGCTCTGCCTATCC-3’ | 5’- CATCTCAGATCCCGAAGGAA-3’ | 55 | 32 | 819 | 4.199 |
| MMP-11 | 5’-GGGGATGTCCACTTCGACTA-3’ | 5’-CAGTGGGTAGCGAAAGGTGT-3’ | 50 | 32 | 165 | 308 |
| MMP-12 | 5’-ACAGATGATGGACCCTGGTT-3’ | 5’-AGAGTCAAGCAAGAATGGACAA-3’ | 58 | 32 | 392 | 1.337 |
| MMP-13 | 5’-AACATCCAAAAACGCCAGAC-3’ | 5’-GGAAGTTCTGGCCAAAATGA-3’ | 57 | 32 | 166 | 1.117 |
| MMP-14 | 5’-GAGCTCAGGGCAGTGGATAG-3’ | 5’-AGCAGATGACCCCATTTGAC-3’ | 59 | 32 | 798 | 1.137 |
| MMP-15 | 5’-AGGAGACACAGCGTGGAGAC-3’ | 5’-TTGCAGTAAAGCAGGACACG-3’ | 56 | 32 | 514 | 1.893 |
| MMP-16 | 5’-GACATGCTCTGGGATTGGAG -3’ | 5’-TCATTTTTCCTTGGGTCAGC -3’ | 56 | 32 | 217 | 2198 |
| MMP-17 | 5’-GGAGCTGTCTAAGGCCATCA-3’ | 5’-CGACAGGTTCCTCTTGTTCC-3’ | 56 | 32 | 190 | 474 |
| MMP-19 | 5’-CAGCCTCGTTGTGGCCTAGA-3’ | 5’-ACCAGCCTGCACCTCTTGGA-3’ | 55 | 32 | 207 | 430 |
| MMP-20 | 5’-CGACAATGCTGAGAAGTGGA-3’ | 5’-ATCTTTGGGGAGGTGGAATC-3’ | 57 | 32 | 169 | 975 |
| MMP-21 | 5’-GACGACGACGAGCACTTCAC-3’ | 5’-TTTCCTGTCTGACCAGTCCA-3’ | 53 | 32 | 180 | 431 |
| MMP-23 | 5’-TGGGACCACTTCAACCTCAC-3’ | 5’-CGTGTTGTGAGTGCATCAGG-3’ | 55 | 32 | 412 | 938 |
| MMP-24 | 5’-GAACCTGTGGGCAAGACCTA-3’ | 5’-TGACAACCAGAAACTGAGCG-3’ | 52 | 32 | 214 | 2.650 |
| MMP-25 | 5’-CCATTATGAGGCCCTTCTACC-3’ | 5’-TAGGTCTTCCCGTTCTGTGG-3’ | 55 | 32 | 541 | 1.422 |
| MMP-26 | 5’-GATATGAAGCCATCCGCAGT-3’ | 5’-GCTGGAAGGTTCTAGGGTCG-3’ | 58 | 32 | 378 | 1.470 |
| MMP-27 | 5’-TTGTTTCTTGTGGCTGCTCA-3’ | 5’-GCTAAGCCAAAGGAACCCAC-3’ | 53 | 32 | 194 | 376 |
| MMP-28 | 5’-CACCTCCACTCGATTCAGCG-3’ | 5’-AAAGCGTTTCTTACGCCTCA-3’ | 57 | 32 | 208 | 376 |
| PBGD | 5’- GGAAGTGCGAGCCAAGGACCAGGA -3’ | 5’- AGGGACATGGATGGTAGCCTGCAT -3’ | 60 | 28 | 241 | 598 |

Tm = annealing temperature, bp = base pair
